# Malignant Course of a Metastatic Melanoma During Pregnancy: A Case Report

**DOI:** 10.4021/wjon285w

**Published:** 2011-04-09

**Authors:** Cugati Goutham, Jain Pradeep Kumar, Pande Anil, Symss Nigel Peter, Ramamurthi Ravi, Vasudevan Mathabushi Chakravorthy

**Affiliations:** a Dr. Achanta Lakshmipathi Neurosurgical Center, Post Graduate Institute of Neurological Surgery, Voluntary Health Services Hospital, Chennai - 600113, India

**Keywords:** Cerebral metastasis, Malignant melanoma, Metastatic melanoma, Pregnancy, Spinal metastasis

## Abstract

Cutaneous melanoma can metastasize to any organ, including brain and spinal cord. A 27-year-old lady, four months after conception presented with generalized seizures and was diagnosed to have subarachnoid hemorrhage. Further investigation did not reveal aneurysm. She underwent right ventriculo-peritoneal shunt for hydrocephalus and MTP for unprotected radiation from CT scan. Six weeks later she came to our institution with symptoms of dorsal compression. Imaging showed multiple intradural extramedullary spinal lesions at D3-D4, D8 and D10-D11. Surgical excision of the lesions was done and histopathology was consistent with metastatic deposits from malignant melanoma which was confirmed by immunohistochemistry studies also. Her conscious level deteriorated on the second postoperative day and CT scan showed multiple small tumor emboli with evidence of right temporal bleed and diffuse cerebral edema. In spite of aggressive treatment she could not be saved. This reported case concludes that pregnancy aggravates the clinical course of metastatic melanoma.

## Introduction

Cutaneous melanoma is known to have the capacity to metastasize to virtually any organ [1]. Cerebral metastasis is seen in 10% and symptomatic spinal metastasis is seen in 0.5% of the patients with cutaneous melanoma [2, 3]. We report a case of malignant melanoma with cranial and spinal metastasis which took a fulminant course during pregnancy and proved to be fatal.

## Case Report

**Figure 1:**
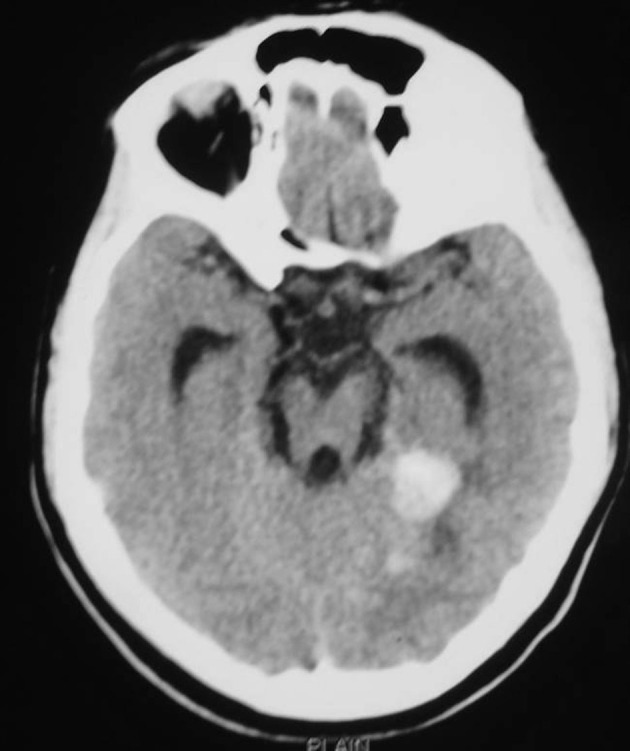
CT scan of the brain showing diffuse subarachnoid hemorrhage and foci of bleed in the right medial posterior temporal region.

A 27-year-old lady married one and a half years before and conceived 4 months before was having a normal growth of the fetus as per the ultrasound report. At 2 months of gestational age she had sudden onset of severe global headache associated with vomiting and one episode of generalized seizures for which she sought treatment in another neurosurgical center. CT scan of the brain revealed diffuse subarachnoid hemorrhage and foci of bleed in the right medial posterior temporal region (Fig. 1). It was suspected to be an aneurysmal bleed and CT angiogram was done which did not show any evidence of intracranial aneurysm. As there was early hydrocephalus she underwent right VP shunt. Gynaecological opinion was sought and in view of unprotected radiation and increased risk of congenital fetal malformations, she also underwent medical termination of pregnancy.

**Figure 2:**
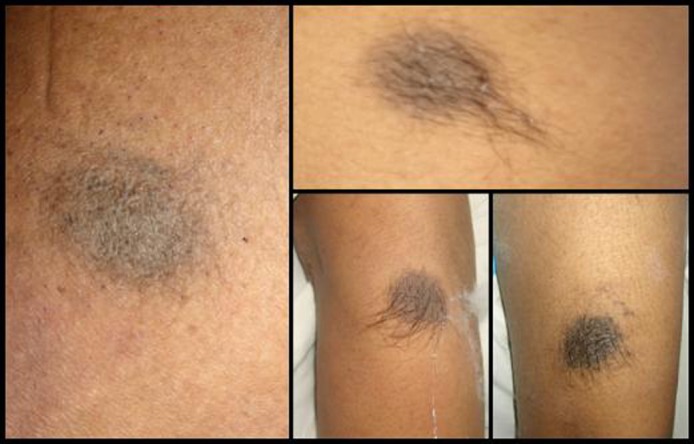
Pictures showing multiple nevi over the various parts of the body.

Six weeks later she developed rapidly progressive weakness of the lower limbs and urinary retention for which she came to our institute. On examination she was conscious and oriented. Fundus was normal with no evidence of intraocular melanoma. Her motor power, sensations and reflexes were normal in the upper limbs. Motor power was 2/5 in right lower limb and 4/5 in left lower limb. There was no obvious sensory deficit. Her lower limb reflexes were brisk and plantars were extensor. She had multiple nevi over the body distributed over the face, back, thighs, legs, foot, sole or arms (Fig. 2). The nevi over the back and the sole had increased in the size and pigmentation in the recent past. Cardiovascular system, respiratory system and per abdominal examination were normal. No palpable lymph nodes felt. There was no spinal tenderness or deformity.

**Figure 3:**
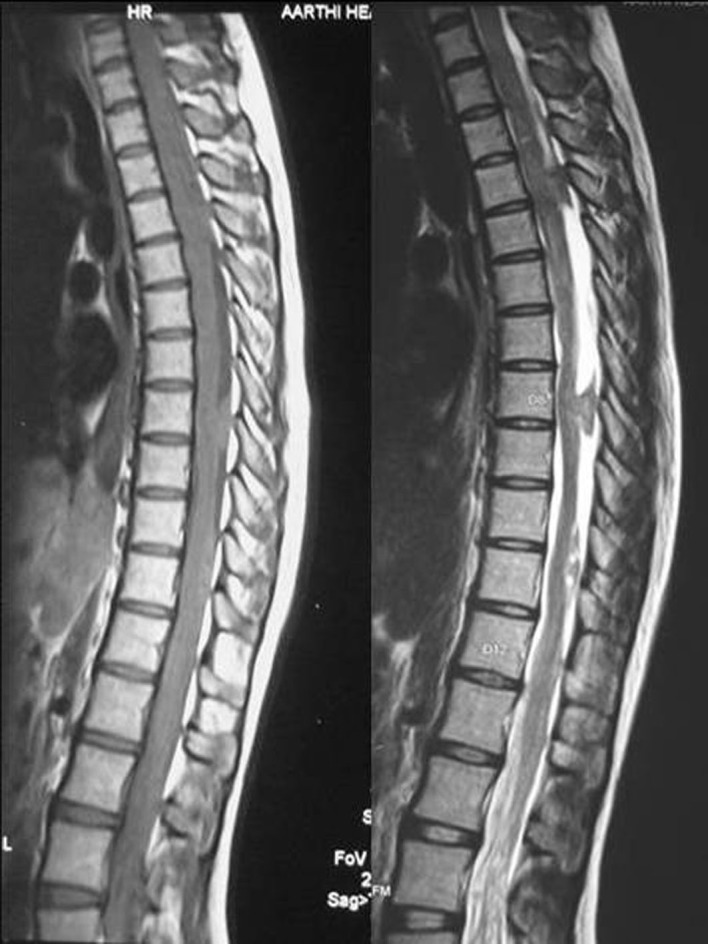
MRI scans of the spine (sagittal view) showing multiple intradural extramedullary lesions at D3-D4, D8 and D10-D11 which were isointense on T1W and hyperintense on T2W.

MRI scan of the spine revealed multiple intradural extramedullary spinal lesions at D3-D4, D8 and D10-D11. All the lesions were isointense on T1W and hyperintense on T2W with no significant enhancement with the contrast (Fig. 3).

**Figure 4:**
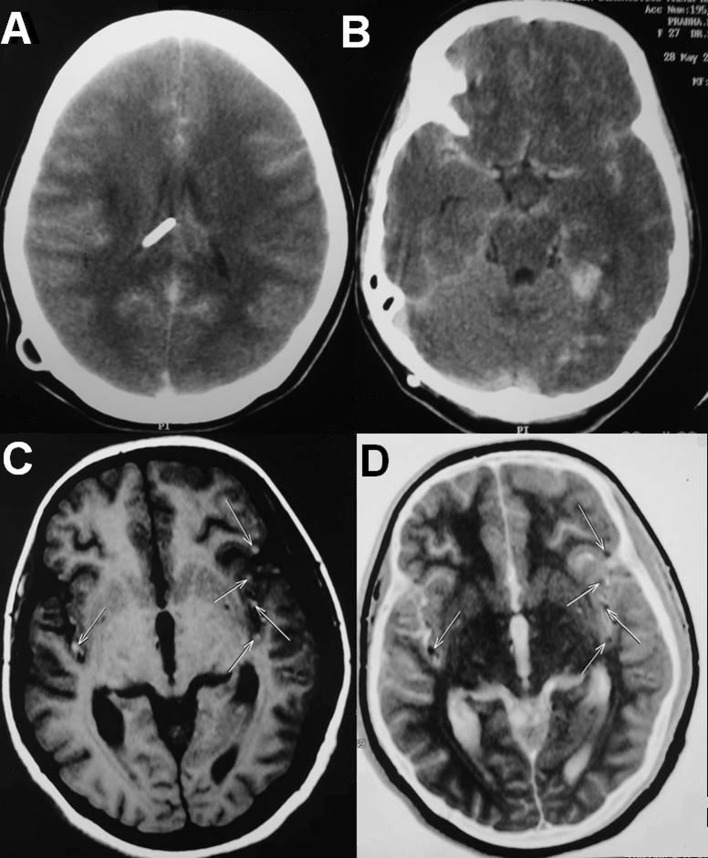
(A, B) Repeat CT scans of the brain showing multiple small tumor emboli with evidence of right temporal bleed and diffuse cerebral edema. (C, D) MRI of the brain showing the tumor emboli on both the hemispheres in middle cerebral artery territory.

She underwent D4 and D8 laminectomies and total excision of the lesions at two levels. The lesions were intradural extramedullary dirty black in color, soft in consistency, easily separable from the spinal cord and were attached to the overlying dura. HPE and immunohistochemistry were consistent with metastatic deposits of malignant melanoma. Postoperatively she improved in her paresis by one MRC grade. On second postoperative day she started having frequent episodes of irrelevant speech and the next day she became drowsy with respiratory distress. She was intubated and ventilated. Repeat CT scan of the brain showed multiple small tumour emboli with evidence of right temporal bleed and diffuse cerebral edema (Fig. 4A, B). The tumor emboli were clearly made out in the MRI of the brain (Fig. 4C, D). She was given aggressive antiedema measures in spite of which she expired on the fourth postoperative day as a result of malignant cerebral edema.

## Discussion

Melanoma accounts for only 4% of all skin cancers, however, it is responsible for more than 74% of skin cancer deaths. Invasive melanoma has a higher female predilection from birth to age 39 years [4]. Due to advances in the cancer therapy there has been increased incidence of symptomatic spinal metastasis from melanoma. In the study conducted by Arif Aladag et al at Aderson cancer center, out of the 144 patients with spinal metastasis from melanoma, only five patients had symptomatic leptomeningeal spread. The survival rate of patients with spinal metastasis was nine months [2]. Other similar studies also showed a poor prognosis with a survival of less than one year with patients with metastatic melanoma [5, 6].

For many years, there has been controversy regarding the correlation of female reproductive factors and hormones with the development and outcome of malignant melanoma. In studies conducted on pregnant women with localized melanoma without metastasis, there was no significant increased risk of death as compared with the non-pregnant controls and the prognosis was excellent [7, 8]. In studies conducted on pregnant women with metastatic melanoma the disease free interval was shorter as a result of decreased duration for nodal metastasis [9]. A population-based study conducted by O’Meara et al did not find supporting data to conclude that pregnancy is associated with more advanced stage, thicker tumors, increased metastasis to lymph nodes, or a worsened survival [8].

In this reported case though the patient was having multiple nevi since birth, the change in the pattern of the nevi was noted only after she conceived but medical attention was not sought. She developed subarachnoid hemorrhage due to cerebral metastasis 8 weeks after she was conceived. Six weeks later she became symptomatic for the spinal lesions. The metastasis was so rapid that within four days of the spinal surgery, she developed malignant cerebral edema secondary to the cerebral metastasis and expired.

In conclusion, this reported case supports the fact that pregnancy aggravates the clinical course of metastatic melanoma. One must be very cautious when treating the pregnant patients with melanoma. We also await more studies from Indian population showing the effect of pregnancy on clinical course of localized, malignant and metastatic melanoma.
